# Vitamin B_12_ and transcobalamin in bovine milk: Genetic variation and genome-wide association with loci along the genome

**DOI:** 10.3168/jdsc.2020-0048

**Published:** 2021-03-12

**Authors:** Grum Gebreyesus, Nina Aagaard Poulsen, Mette Krogh Larsen, Lotte Bach Larsen, Esben Skipper Sørensen, Christian Würtz Heegaard, Bart Buitenhuis

**Affiliations:** 1 Center for Quantitative Genetics and Genomics, Aarhus University, Blichers Allé 20, PO Box 50, DK-8830 Tjele, Denmark; 2 Department of Food Science, Aarhus University, Agro Food Park 48, DK-8200 Aarhus N, Denmark; 3 Arla Foods Amba, Mæalkevejen 4, DK-6920 Videbæk, Denmark; 4 Molecular Nutrition, Department of Molecular Biology and Genetics, Aarhus University, Gustav Wieds Vej 10, DK-8000 Aarhus C, Denmark

## Abstract

In human nutrition, bovine milk is an essential source of bioavailable vitamin B_12_ and B_12_-binding proteins, including transcobalamin. In this study, we estimated genetic parameters for milk content of vitamin B_12_ and transcobalamin using milk samples from 341 and 663 Danish Holstein cows, respectively. Additionally, we conducted whole-genome association analysis to identify SNP and genes associated with vitamin B_12_ and transcobalamin. Our results indicated moderate to high heritability for vitamin B_12_ (0.37 ± 0.18) and transcobalamin (0.61 ± 0.13) content in the Danish Holstein. With a significance threshold of –log_10_*P*-value > 5.87, significant associations were detected between SNP in *Bos taurus* autosome (BTA)17 and the log-transformed transcobalamin content of milk; no significant association was detected for vitamin B_12_. The significant region in BTA17 was imputed to full sequence for further fine mapping, and the SNP with the most significant associations to transcobalamin were assigned to the transcobalamin 2 (*TCN2*) gene.

## Highlights

• High heritability (0.61 ± 0.13) was found for milk transcobalamin content in Danish Holstein.
• Moderate heritability (0.37 ± 0.18) was found for vitamin B12 content in Danish Holstein.
• Twenty-eight SNPs were detected with strong association to the milk content of transcobalamin.
• A strong QTL region was detected for transcobalamin on BTA17 (71.71–71.79 Mbp).

## Graphical Abstract

**Figure:**
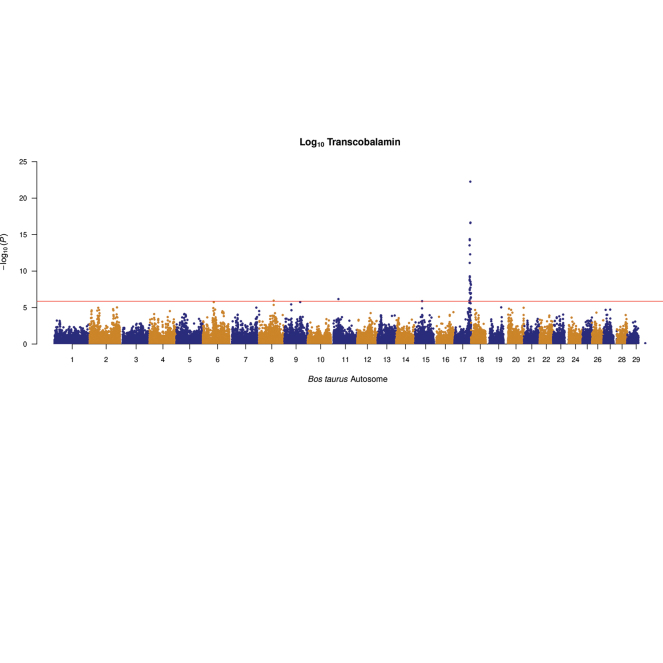
**Summary:** This study reports significant associations between the log-transformed transcobalamin content in the Danish Holstein milk and 28 single nucleotide polymorphisms (SNPs) across Bos taurus autosomes (BTA). Of these 28 significantly associated SNPs, 24 were on BTA17, where strong associations were detected, with −log_10_(P-value) up to 62.93. The QTL region on BTA17 spanned between 71.71 and 71.79 Mbp.

In human nutrition, milk and dairy products are important sources for several vitamins, including cobalamin (vitamin B_12_; Rooke et al., 2010). The primary origin of water-soluble vitamins, including B_12_, is through microorganism biosynthesis in the rumen (Schwab et al., 2006) and products from ruminants, such as milk and meat, which are generally rich in B_12_ vitamins. Studies have established links between vitamin B_12_ deficiency and some serious disorders in humans, including neurodegeneration and anemia (Hahn et al., 1988; Green and Lindsay, 2017). Humans are ultimately dependent on animal sources of vitamin B_12_, and deficiency is caused by inadequate intake, inadequate bioavailability, or malabsorption. Deficiency can affect individuals at all ages but particularly elderly individuals and vegetarians (Song et al., 2010; Green and Lindsay, 2017).

Studies indicate that vitamin B_12_ in milk from cows has a high bioavailability (Matte et al., 2014). Bovine bulk milk contains approximately 2 to 6 μg/L of vitamin B_12_ (Duplessis et al., 2016); therefore, 1 L of milk contains more than the current recommended dietary allowance for adults (2.4 μg/d; Institute of Medicine, 1998). In milk from cows, B_12_ is evenly distributed between the whey and the casein micelle fraction, bound to transcobalamin (**TC**) in the whey, and via coordination to histidine residues of the caseins, respectively (Fedosov et al., 2018, 2019). In humans, TC functions primarily as a circulatory protein, which binds B_12_ following its absorption and delivers it to peripheral tissues via the receptor CD320. Recently, we found that cow milk-derived TC–B_12_ complex was more effective at stimulating receptor-mediated passage of B_12_ across polarized monolayers of human intestinal epithelial (Caco-2) cells than human TC–B_12_ complex (Juul et al., 2019). Clinical studies have shown that 8 wk of daily intake of whey protein isolate improved biomarkers of B_12_ status in elderly Australians with subclinical B_12_ deficiency and, more recently, long-term daily whey powder intake was shown to reinforce B_12_ status in healthy elderly Danes (Dhillon et al., 2017; Greibe et al., 2020). Furthermore, it has been reported that whey powder supplement provided over 4 wk was as efficient as synthetic B_12_ vitamin pills in improving biomarkers of B_12_ deficiency in lactovegetarians (Naik et al., 2019).

Despite the acknowledged value of bovine milk as a B_12_ source (Rooke et al., 2010), very few studies have documented the drivers for B_12_ and TC content variation in milk within and across bovine breeds. Rutten et al. (2013) quantified B_12_ in 544 first-lactation Dutch Holstein-Friesian cows and found an average content of 4.40 mg/L (range 1.0 to 12.9 mg/L). The study estimated a moderate heritability of 0.37, suggesting that vitamin B_12_ content in milk could be altered through genetic selection. A genome-wide association study identified 68 significant SNP associated with B_12_, but none of these was associated with genes involved in B_12_ transport (Rutten et al., 2013).

The aim of this study was to determine the contents of B_12_ and TC in milk from Danish Holstein cows and estimate genetic influence on these traits. An additional aim was to identify QTL associated with the variability in milk B_12_ and TC contents. To our knowledge, this is the first study to screen B_12_ and TC at the same time in milk from a large number of cows.

Morning milk samples were collected from 663 Danish Holstein cows in 21 herds in Denmark, as described by Gebreyesus et al. (2017). Of the collected milk samples, only 341 were used to quantify B_12_, whereas TC was quantified in all samples.

All procedures to collect the samples followed the protocols approved by the National Guidelines for Animal Experimentation and the Danish Animal Experimental Ethics Committee. Milk sampling was restricted to routine on-farm procedures that did not cause any inconvenience or stress to the animals; hence, no specific permission was required.

Total B_12_ was measured by the standard procedure for the Advia Centaur CP System (Siemens Healthcare Diagnostics), using hydroxo-B_12_ as standard. The endogenous B_12_ was extracted from the milk samples as previously described (Fedosov et al., 2019).

The in-house bovine TC ELISA protocol was performed as previously described (Fedosov et al., 2019). A custom-made polyclonal rabbit antibody and a monoclonal antibody, both raised against recombinant bovine TC, were used as capture and detect antibodies, respectively. Recombinant bovine TC was used as calibrator.

Genotyping was performed as described in Buitenhuis et al. (2013). In short, genomic DNA was extracted from ear tissues of 663 Danish Holstein cows. In total, 341 animals were genotyped with the BovineHD BeadChip (Van Tassell et al., 2008), whereas the remaining 322 animals were genotyped with the Bovine50K BeadChip (Illumina Inc.). The genotyping was accomplished using an Illumina Infinium II Multisample assay device. iScan and Beadstudio version 3.1 software (Illumina Inc.) were used for scanning and analysis of the SNP chips. Quality parameters for the selection of SNP were as outlined by Buitenhuis et al. (2013), and individuals with average GenCall scores <0.65 were excluded, following Teo et al. (2007). Based on the overlap between the 2 SNP chips, 37,458 SNP were used to calculate the genomic relationship matrix and the initial association analysis. Genomic relationship matrix was calculated following method 1 of VanRaden (2008).

Genotype on BTA17 was imputed to full sequence in a 2-step procedure, as described in detail in Gebreyesus et al. (2016). First, the group of cows genotyped using the Bovine50K chip were imputed to the BovineHD (777K) level using a reference of 3,383 animals, including the 341 cows from the current study. In this step, only the 50K SNP that passed quality control (i.e., minor allele frequency >0.05 and GenCall scores ≥0.65) were used in the target population. Subsequently, the true and imputed high-density data for both groups of cows were merged, and imputation was undertaken to the whole-genome sequence level for BTA17 using a reference of 1,228 animals from run4 of the 1,000 Bull Genomes project (Daetwyler et al., 2014). In both steps, data sets of different densities were pre-phased with Beagle 4 r1398 (Browning and Browning, 2013) and imputed using IMPUTE2 v2.3.1 (Howie et al., 2011). After imputation, a total of 391,026 variants were available on BTA17 for the fine-mapping study. The SNP positions were based on the *Bos taurus* genome assembly UMD 3.1 (Zimin et al., 2009).

Records for both B_12_ and TC were log-transformed for the genetic analyses following tests for normality. The REML approach in DMU (Madsen and Jensen, 2007) was used to estimate the genetic parameters and variance components using the following model in the analysis

[1] $$Y_{ikjl}=\mu +herd_{i}+parity_{j}+b_{1}DIM_{k}+b_{2}e^{-0.05\times DIM_{k}}+animal_{l}+e_{ijkl},$$

where *Y_ijkl_* represents the phenotype of individual *l* in herd *i* and parity *j, μ* is the overall mean, *herd_i_* (*i* = 1, 2, …, 21 for TC, and *i* = 1, …, 3 for B_12_) and *parity_j_* (*j* = 1, 2, …, 5) are fixed effects; *b*_1_ and *b*_2_ are regression coefficients for *DIM_k_* and $e^{-0.05\times DIM_{k}}$, respectively, where *DIM_k_* is a covariate of days in milk (d 4 to 877), and $e^{-0.05\times DIM_{k}}$ is the Wilmink adjustment of DIM (Wilmink, 1987); *animal_l_* is a random additive genetic effect of animal *l* based on the genomic relationship matrix **G**; and *e_ijkl_* is the random residual effect.

Milk samples were collected once on each farm and during the same season across farms; therefore, a season effect was not fitted into the model.

Heritability (*h*^2^) estimate was defined as

[2] $$h^{2}=\frac{\sigma_{a}^{2}}{\left(\sigma_{a}^{2}+\sigma_{e}^{2}\right)},$$

where $\sigma_{a}^{2}$ is the genetic variation, and $\sigma_{e}^{2}$ is the residual variation based on univariate analyses.

The association analysis was performed based on an extension of the linear model [1] with an allele substitution effect (*b*_3_) and a covariate *SNP_m_*, indicating whether a SNP was heterozygous (1) or homozygous (0, 2). The effect of the SNP was tested using a Wald test with a null hypothesis of H_0_: *b* = 0. For both traits, SNP effects were declared significant if the corresponding −log_10_ *P*-value was >5.87 (based on genome-wide Bonferroni correction). Additional association analysis was implemented to fine-map QTL regions detected in BTA17 using imputed sequence variants and using a similar model.

Table 1 presents descriptive statistics and genetic parameters for milk B_12_ and TC content in the Danish Holstein. The mean B_12_ content was 3.93 µg/L, with a coefficient of variation of 41%. Accordingly, B_12_ content in the Danish Holstein varied from 1.06 to 10.28 µg/L. The content and ranges found in this study for B_12_ are in line with the findings by Rutten et al. (2013), and with levels generally reported from bovine milk (Akins et al., 2013; Duplessis et al., 2016).

**Table 1:** Descriptive statistics and genetic parameters for milk vitamin B_12_ and milk transcobalamin contents in milk from Danish Holstein cows^1^

Variable	Mean	CV (%)	σ^2^_a_ (SE)	σ^2^_e_ (SE)	*h*^2^ (SE)
B_12_ (μg/L)	3.93	41.0	0.008 (0.090)	0.014 (0.118)	0.37 (0.18)
Transcobalamin (pmol/L)	557.00	81.0	0.036 (0.190)	0.023 (0.151)	0.61 (0.13)

1 σ^2^_a_ = genetic variation, σ^2^_e_ = residual variation, and *h*^2^ = heritability; genetic and residual variation and heritability were estimated based on the log-transformed data.

For TC, the mean content was 577 pmol/L, and the value varied from 96 to 4,672 pmol/L, with a relatively higher coefficient of variation (81%). To our knowledge, TC has never been evaluated in such a large number of milk samples from individual cows, although the mean value found here is in line with those reported for a pooled sample of milk from Danish Holstein cows (Fedosov et al., 2019).

Genetic variance explained a substantial part of the variation, and estimated heritabilities were moderate to high for B_12_ (0.37 ± 0.18) and TC (0.61 ± 0.13), respectively. The heritability estimate found for B_12_ in this study was similar to the estimate for the Dutch Holstein-Friesian (*h*^2^ = 0.37; Rutten et al., 2013) but higher than estimates reported for the Scottish Holstein-Friesian (*h*^2^ = 0.10; Denholm et al., 2019).

Performing a GWAS on the log-transformed data revealed no significant SNP for B_12_ (Figure 1). This is in contrast to the findings of Rutten et al. (2013), which reported 68 SNP that showed significant association with milk B_12_ content in the Dutch Holstein-Friesian. This could be partly explained by the stringent significance threshold used in this study (−log_10_ *P*-value = 5.87) compared with the less stringent significance threshold used in the study of Rutten et al. (2013) (−log_10_ *P*-value = 3.0).

**Figure 1:**
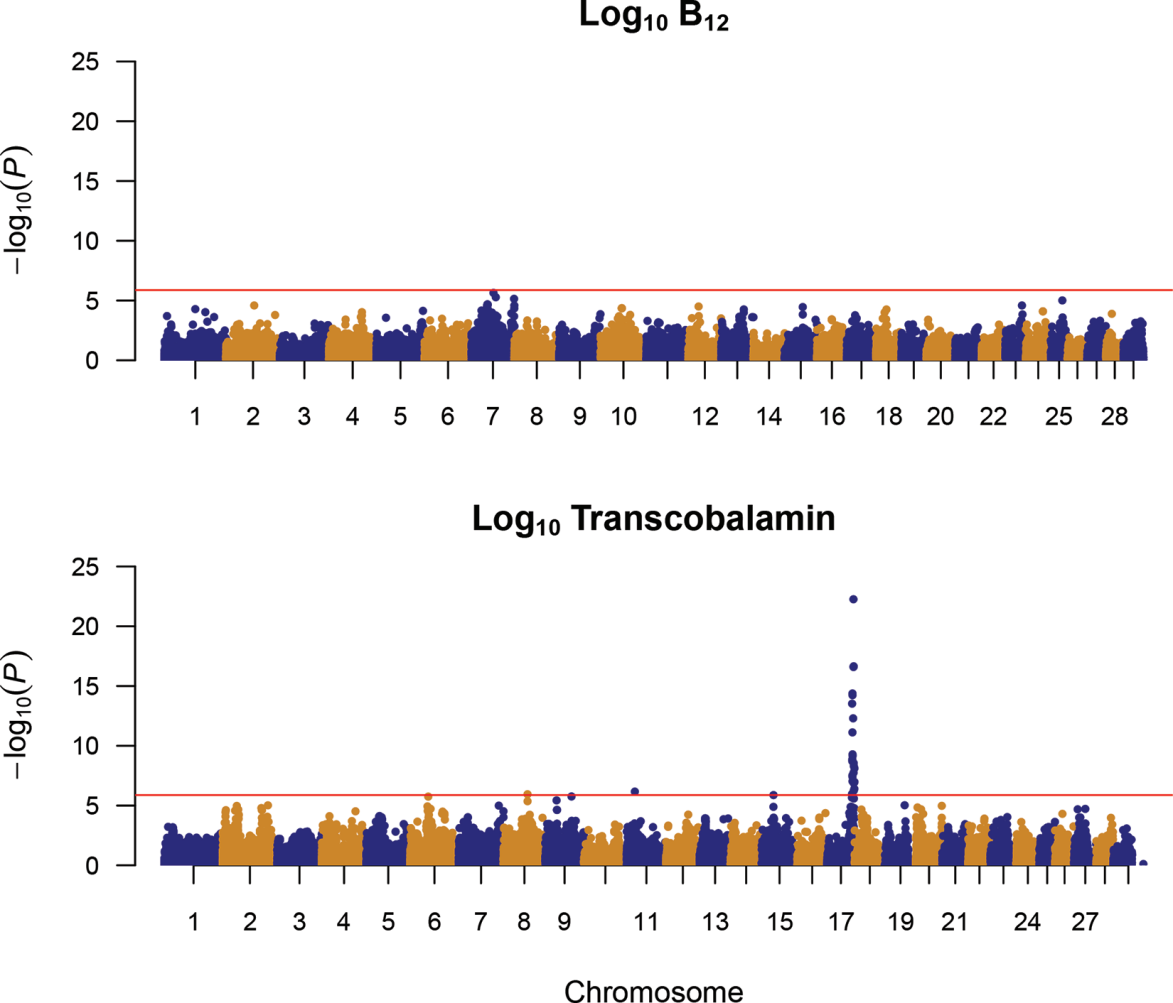
Manhattan plot for association analysis of log_10_ B_12_ (top) and log_10_ transcobalamin (bottom) using 50K genotype data.

In contrast, 28 significant SNP were associated with TC (Figure 1). For the significant SNP, minor allele frequency ranged between 0.07 and 0.49. A very strong QTL was detected for TC on BTA17, and significant associations were detected with 24 SNP. Further fine-mapping using imputed full sequence data showed a QTL region spanning between 71.71 and 71.79 Mbp on BTA17, with the most significant SNP (rs209672492) having a −log_10_ *P*-value of 62.93 (Figure 2). The SNP within this region were assigned to the *TCN2* gene, which encodes transcobalamin, suggesting that the main driver of TC variation relates to variation within the *TCN2* gene or to regulatory elements in close proximity. It should be noted that this study used relatively small sample sizes for both the heritability and GWAS analysis and that results should be interpreted with caution.

**Figure 2:**
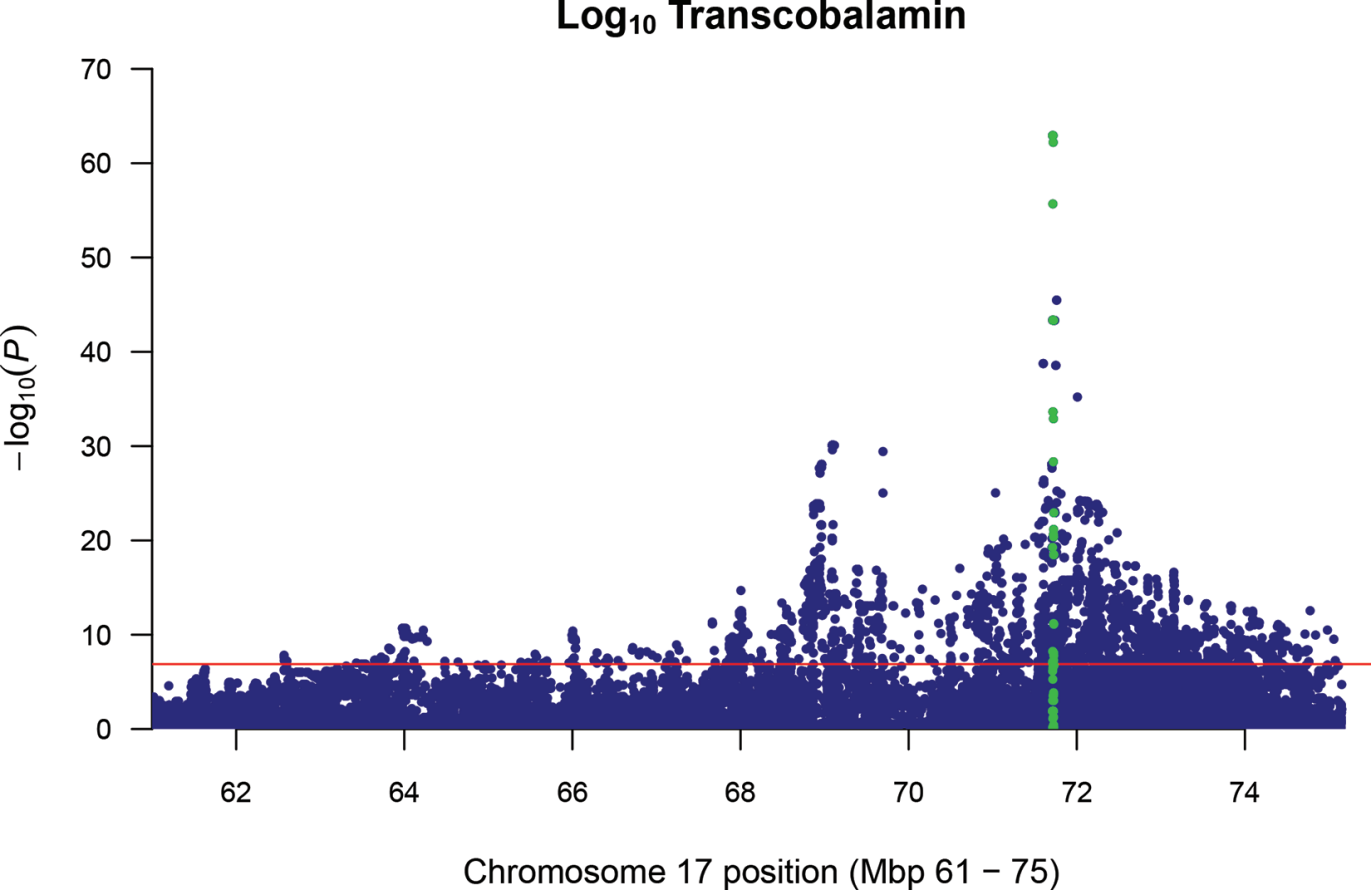
Manhattan plot for the association analysis of log_10_ transcobalamin using imputed full sequence data on BTA17. The SNP within the *TCN2* gene region are highlighted in green.
